# The Smoke Nuisance

**Published:** 1922-10

**Authors:** 


### THE SMOKE NUISANCE.

Our domestic hearth, about which poets have sung from time immemorial, has apparently much to answer for. Mr. Searles-Wood, Vice-President of the Royal Institute of British Architects—who said that for much of the interesting material in his lecture to the Sanitary Inspectors he was indebted to the book, "The Smokeless City," issued by the Lord Mayor of Manchester and Miss Marion Fitz-gerald—asks us all to face the possibility of our cheerful domestic hearth giving way to "central heating." It may, however, be rescued from extinction if the twin problems of fuel economy and smoke abatement can be solved along the lines of development of the combined coke boiler and open fire. Our domestic chimney, says Mr. Searles-Wood, is responsible for three-fourths of the smoke which pollutes the atmosphere of our towns and cities, and more than three-fourths of the damage. The particles that escape as soot are not so completely burnt; they contain a large percentage of tar and acid which attacks and eats into the surface of everything on which it settles. Smoke levies toll on buildings, furniture, curtains, wallpapers, goods in shops, trees, vegetables, personal health and well-being. The damage is difficult to measure, but an interesting calculation has shown that the yearly cost of household washing alone in Manchester would be reduced by £250,000 if that city were as clean as Buxton. And, all the time, 75 or 80 per cent. of the heat of coal used in the fire in the open grate is disappearing up the chimney, and not contributing at all to the comfort of the occupants of the room.

Mr. Searles-Wood derives satisfaction from the friendly rivalry between gas and electricity. Both methods of heating are excellent, but although cheaper than coal for intermittent use, are far and away more expensive for continuous consumption.

With central heating, coke should be used; it is smokeless; little labour is involved, as one fire, warming the whole house, is sufficient; and real economy is secured. The time is opportune for reconsidering our prejudices in favour of the domestic hearth. There are tremendous arrears in house building which have to be made good, providing an unequalled opportunity for taking the right turning in regard to smoke control; the public are becoming alive to the need of economy in every direction, and attuned to the consideration of new methods without prejudice.

There is, it appears, still an extraordinary lack of knowledge as to the relative efficiency of various kinds of heating apparatus, and need for education both of the public and of the manufacturers. There is abundant room for research. The Government Fuel Board are carrying out a most important investigation into the possibility of producing a smokeless solid fuel at reasonable cost, and also giving grants in aid of research. If experiment and research result in our being able, with a clear conscience—because of a clear atmosphere—to retain our cheerful open fire in one room of the house while getting constant hot water supply and constant gentle heat elsewhere in the house, the solution of the domestic smoke difficulty will be a happy one. And if concurrently with this progress the factory chimneys can be made to follow the advice of the wit who, so long ago as the beginning of the nineteenth century, urged that we should

Make our chimneys chew the cud
Like hungry cows, as chimneys should, we may look forward to clear, clean cities, worthy neighbours of our English countryside.

*(Pressure on our space would prevent us from doing justice in this issue to the other excellent papers read at the Conference, such as those on "Milk Studies," by Mr. J. T. Quinton, of Liverpool, and "The Administration of the Law relating to the Sale of Milk," by Mr. Haygarth Brown, of the Ministry of Agriculture. We propose, therefore, to review these papers in our next issue.*—Ed.)

### THE NATIONAL MILK CONFERENCE.

The programme of the National Milk Conference, to be held at the Guildhall, London, on October 16-18, has now been issued. Viscount Astor, the President of the Conference, will deliver the inaugural address at 10 a.m., on Monday, October 16. There will be two sessions each day, from 10 to 1 and from 2.30 to 5.30. The following papers will be read, and will be followed by discussion: October 16 (morning), "The Breed of Cattle in Relation to (1) Quantity, (2) Chemical Composition, (3) Cost of Milk Production" (Mr. James Mackintosh, National Institute for Research in Dairying); "The Effect of Feeding of Dairy Cattle in Relation to the Cost of Milk Production" (Mr. G. H. Garrad, Agricultural Organiser for Kent); (afternoon), "Contagious Abortion and Other Diseases of Cattle" (Sir Stewart Stockman, M.R.C.V.S., Chief Veterinary Officer and Director of Veterinary Research, Ministry of Agriculture); "The Production of Clean Milk" (Mr. F. Arnold Lejeune). October 17 (morning), "Bovine Tuberculosis and its Relation to Man" (Dr. A. Stanley Griffith, Research Bacteriologist to the Medical Research Council); "The Tuberculin Tests" (Capt. S. R. Douglas, Chairman of the Tuberculin Committee, Medical Research Council); (afternoon) "Variation in the Chemical Composition of Milk" (Dr. Charles Crowther); "Handling and Distribution of Milk" (Mr. John Robertson, Medical Officer of Health, Birmingham). October 18 (morning), "Pasteurisation and other Methods of Treating Milk" (Dr. Georges Dreyer, Professor of Pathology in the University of Oxford, and Dr. Charles North, of New York); (afternoon) "The Food Value of Milk and its Care in the Home" (Dr. Robert Hutchison); "Education of the Public and of the Dairy Industry in regard to Milk" (Sir A. Daniel Hall, Chief Scientific Adviser, Ministry of Agriculture). Applications for membership, or for tickets for non-members, should be made to the hon. secretary, Miss H. M. Willans, National Milk Conference, 3 Bedford Square, W.C.1.

